# Infected Mesenteric Cysts: A Rare Cause of Abdominal Pain

**DOI:** 10.7759/cureus.58975

**Published:** 2024-04-25

**Authors:** Abhishek S Satpathy, Kailash C Mohapatra

**Affiliations:** 1 Surgery, Srirama Chandra Bhanja Medical College and Hospital, Cuttack, IND

**Keywords:** hemorrhage, marsupialization, torsion, enterogenous, chylolymphatic, mesenteric cyst

## Abstract

Mesenteric cysts are mostly congenital cysts of varied etiology. They occur twice as often in females than in males. They have varied clinical presentations. Most of them are asymptomatic, and a few present with abdominal mass, abdominal pain, nausea, and vomiting. Ultrasonography and computed tomography (CT) are essential in their diagnosis. These cysts may get complicated due to hemorrhage, torsion, rupture, or infection and may become life-threatening with features of acute abdominal pain and peritonitis. This is a case presentation of a 22-year-old Indian female who came with abdominal pain and was found to have an infected mesenteric cyst on laparotomy.

## Introduction

Mesenteric cysts are mostly congenital cysts of varied etiology. Specifically, only 60% are present during childhood, while the other 40% occur during adulthood, mostly in the fourth decade of life [1]. Mesenteric cysts are rare, with an incidence of one per 140000 people, and they are twice as common in females compared to males. Furthermore, these cysts can be classified into four types: enterogenous, chylolymphatic, hydatid, and traumatic. Most of these types are asymptomatic and identified incidentally during regular imaging, but others have variable levels of symptoms, ranging from abdominal pain with or without vomiting to acute abdominal catastrophe. Overall, patients with mesenteric cysts commonly present with painless, fluctuating swelling near the umbilicus [2]. 

Abdominal ultrasonography and computed tomography (CT) have an important role in the diagnosis of mesenteric cysts. In terms of treatment, complete surgical excision of the cyst, preferably with the laparoscopic approach, is commonly used. However, incomplete excision of cysts, marsupialization, and internal or external drainage are associated with a high risk of recurrence [1].

This study describes a rare consequence of an even rarer anomaly: a mesenteric cyst infection that requires emergency surgery. This manuscript shows the need for marsupialization for a difficult-to-excise cyst, with complete excision having the risk of short bowel syndrome.

This work presents the case of a 22-year-old Indian female who presented with abdominal pain and was found to have an infected mesenteric cyst on laparotomy. 

## Case presentation

**Figure 1:**
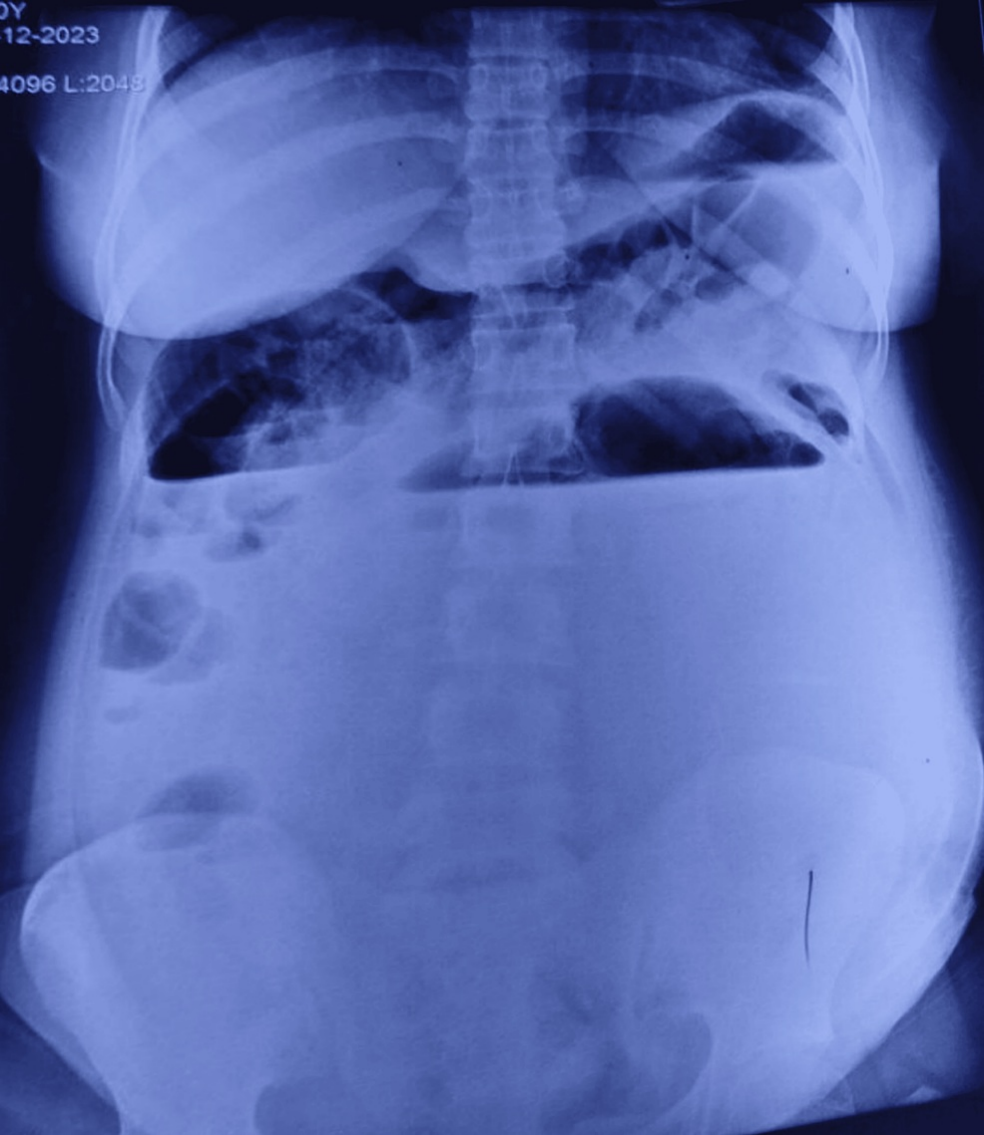
X-ray abdomen, AP view

**Figure 2:**
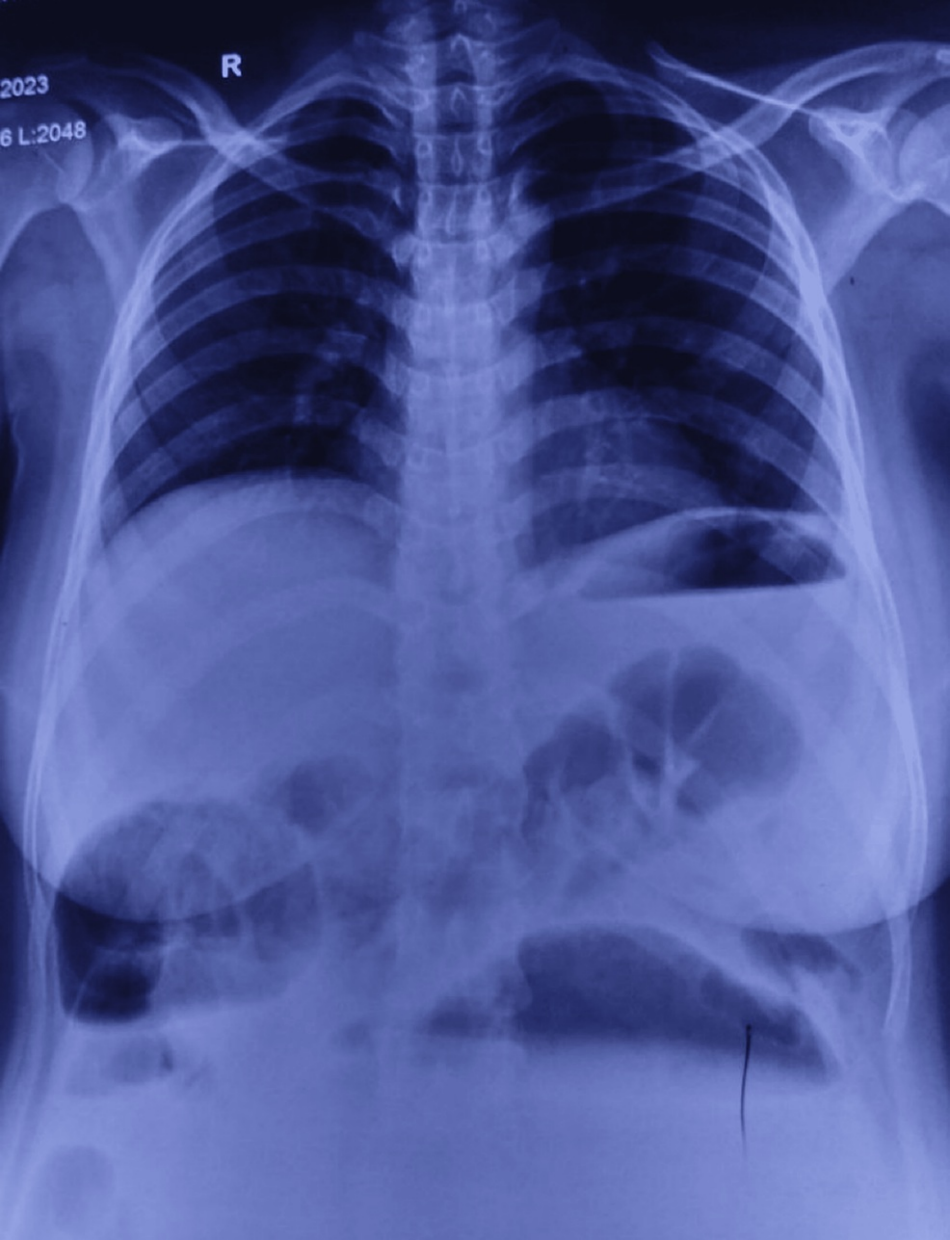
Chest X-ray, PA view

A 22-year-old female patient presented to the hospital complaining of pain and distension of the abdomen for five days. This pain and distention were also associated with intermittent episodes of nausea, vomiting, and fever. The patient was conscious and afebrile, and on examination, the patient had a pulse rate of 108/min and a blood pressure of 100/60 mmHg. The abdominal examination showed distension of the abdomen, which was tense, and on rectal examination, ballooning of the rectum was observed. The X-ray showed multiple air-fluid levels (Figures 1, 2). Sonography of the abdomen revealed a 20x20 cm, well-defined, thick-walled cystic lesion extending from the epigastrium to the pelvis.

**Figure 3:**
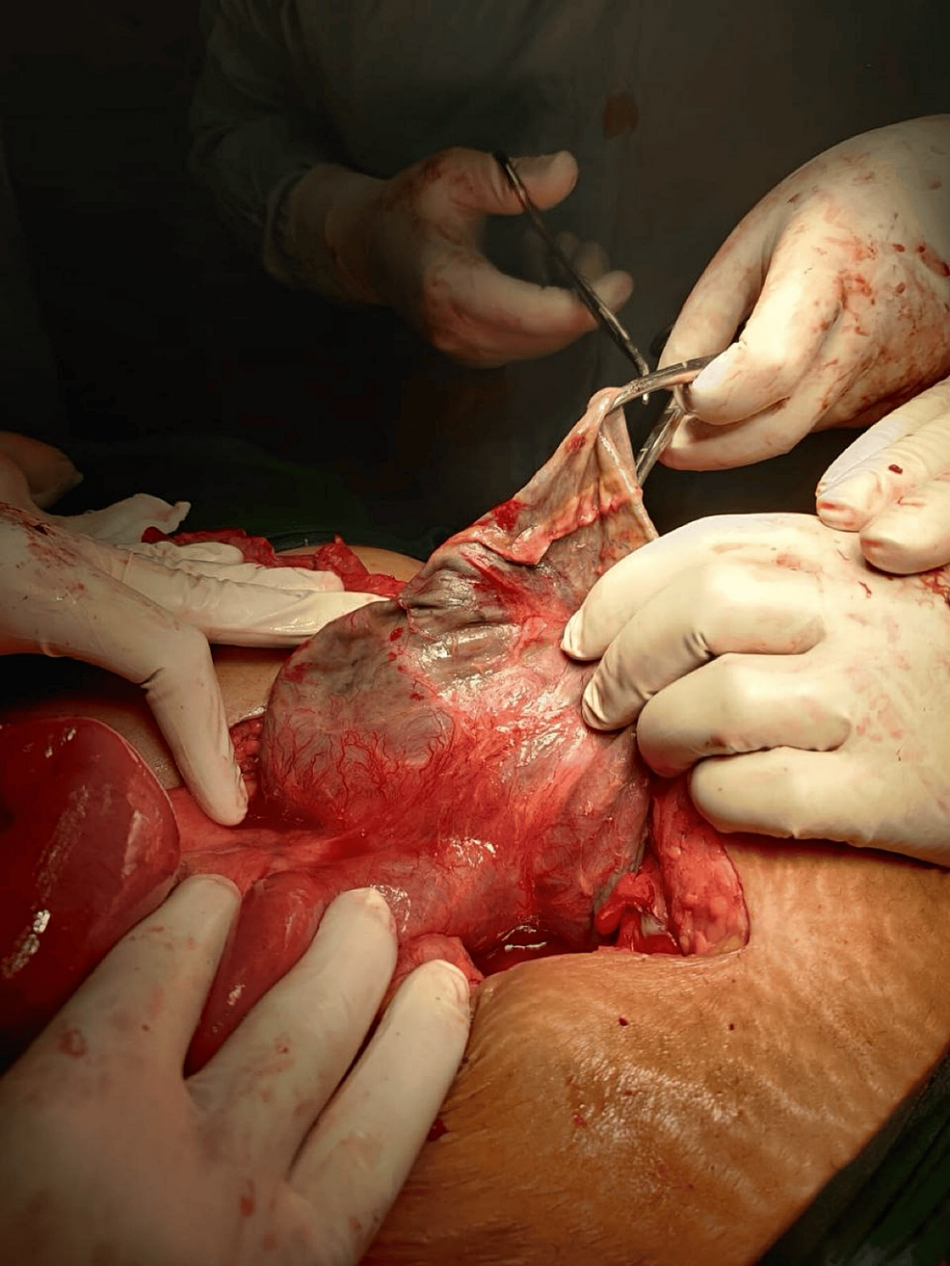
Mesenteric cyst

**Figure 4:**
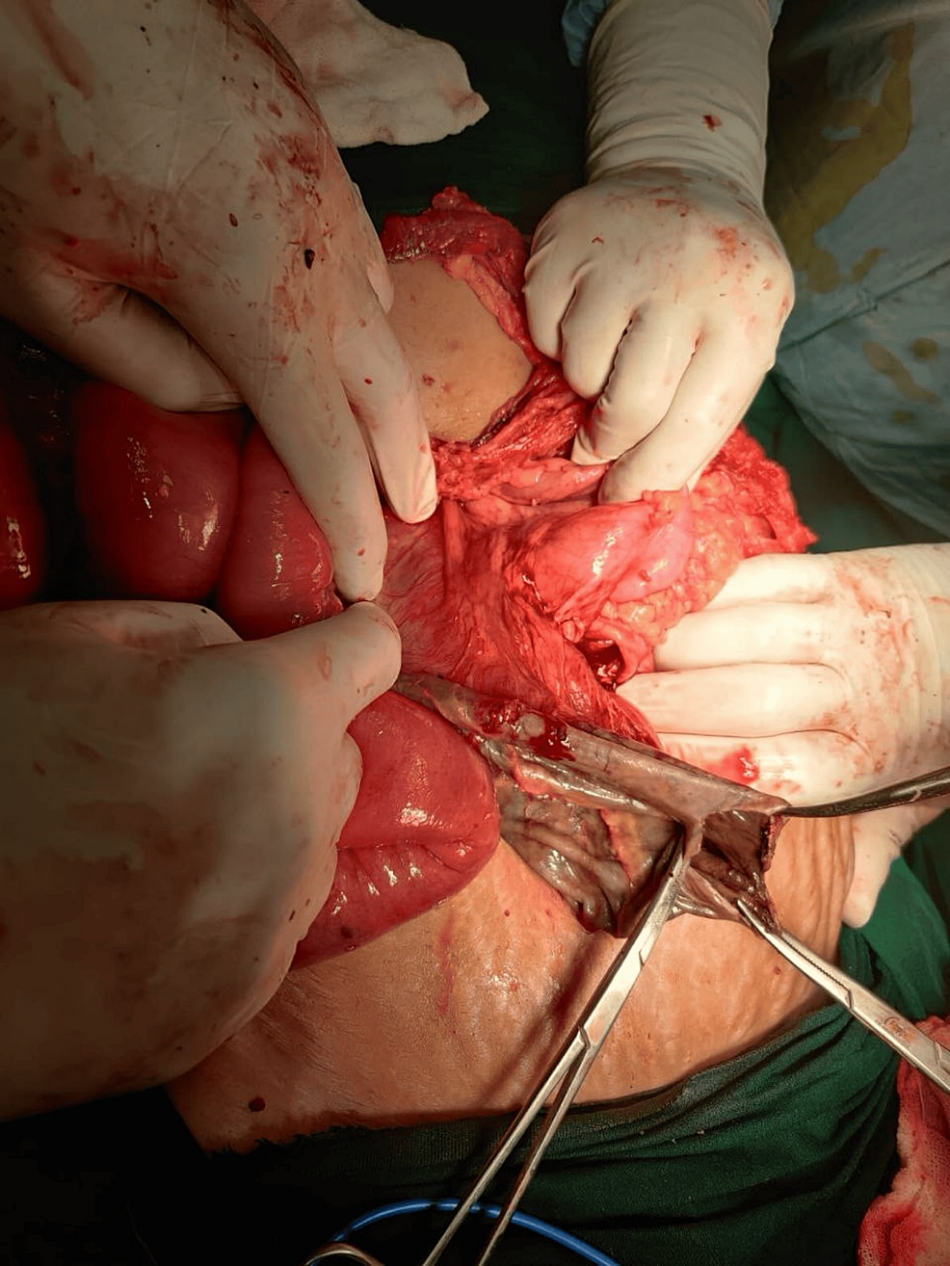
Decompression of the mesenteric cyst and sending the contents for microbiological analysis

**Figure 5:**
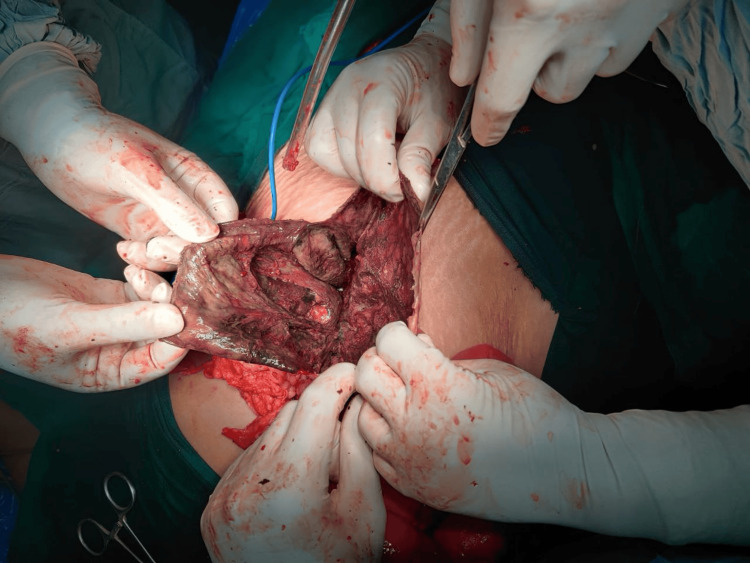
Marsupialization of the mesenteric cyst, resulting in the inner lining of the cyst being observed, and then fixing the wall of the cyst to the mesentery to prevent it from reforming

The patient was scheduled for an emergency laparotomy. The presence of a 20x20 cm mesenteric cyst was confirmed, and when opened, the cyst was found to have hemorrhagic fluid present (Figure 3). The fluid was collected for microbiological analysis (Figure 4). Complete excision of the mesenteric cyst could not be performed as the cyst was attached to the root of the mesentery, and its removal would also involve a large section of the bowel being removed, resulting in short bowel syndrome. Marsupialization of the cyst was completed (Figure 5). A portion of the cyst wall was removed and sent for histopathological evaluation, which identified an infected mesenteric cyst. The pus culture isolate was *Escherichia coli*.

**Figure 6:**
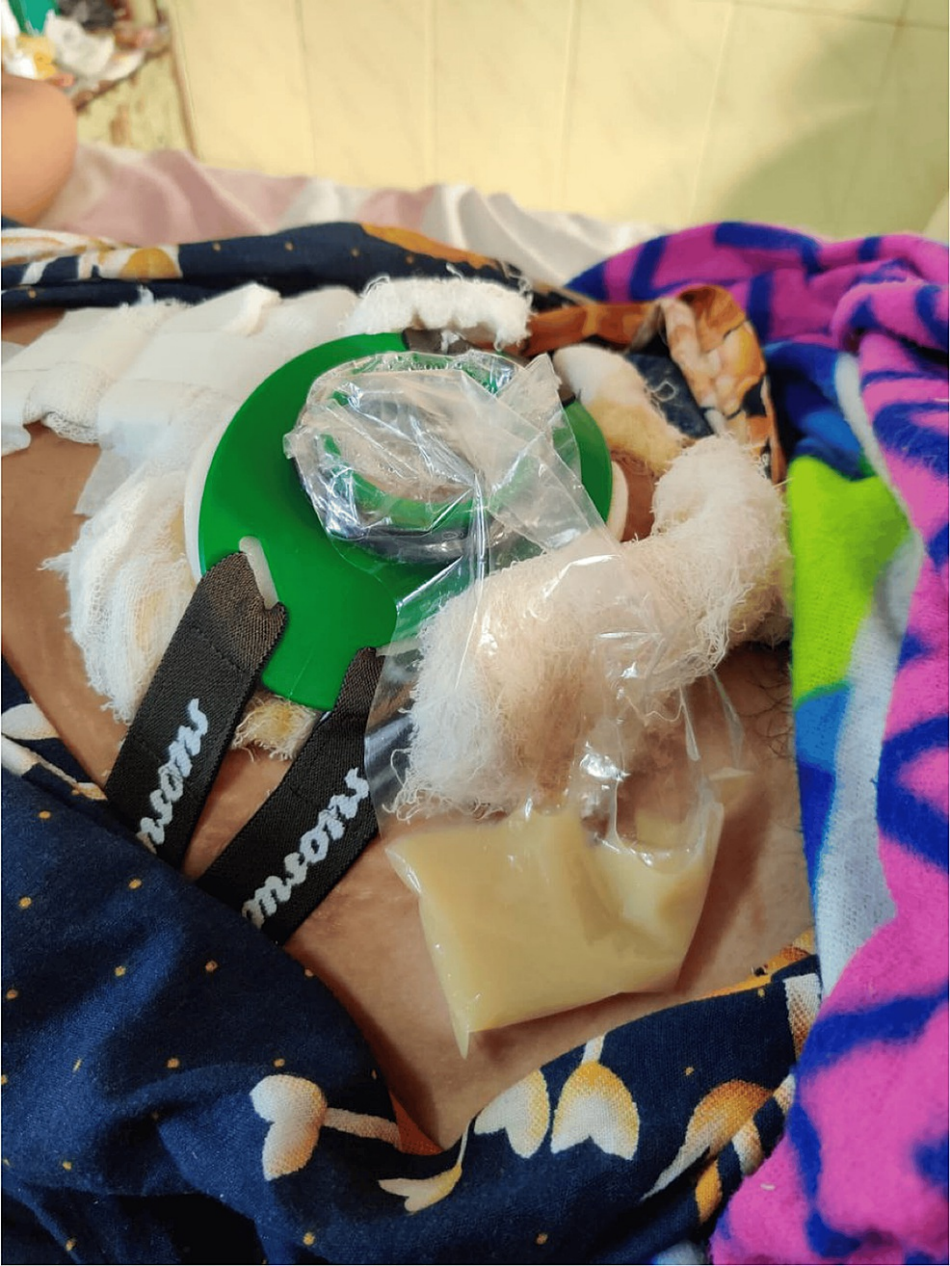
Postoperative wound discharge appearing as purulent fluid in the ostomy bag attached to the main wound to quantify the discharge

**Figure 7:**
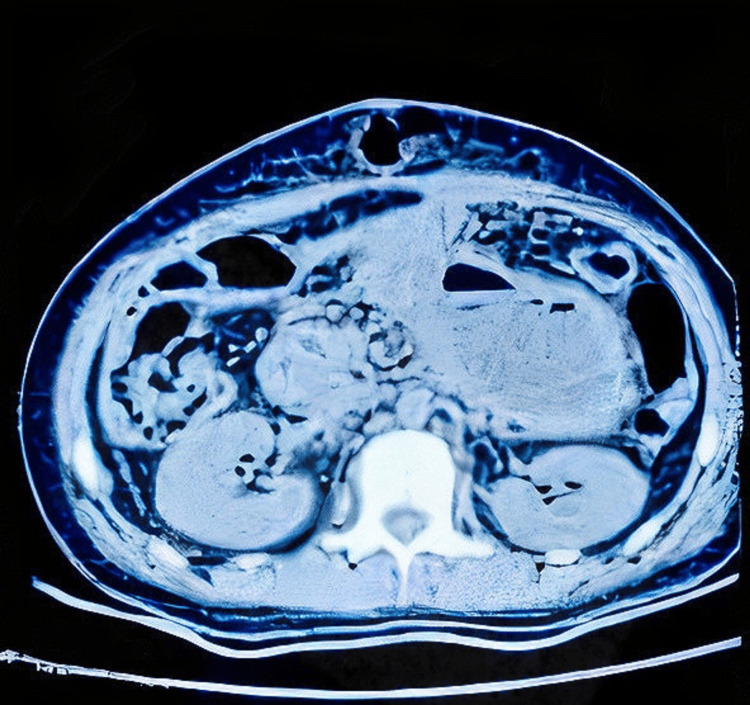
Postoperation CT following main wound discharge showing communication of cyst remnant to the main wound CT, computed tomography

In terms of recovery, the patient received two units of blood following the operation. On postoperative day 5, the bilateral drains were removed, and the patient was allowed oral feeding. The patient recovered uneventfully until postoperative day 10. Subsequently, on postoperative day 10, the patient started having discharge from the midline incision site (Figure 6). The discharge was purulent in nature, and the quantity of discharge was approximately 25 mL per day. A CT scan showed the presence of an irregular, thick-walled cystic lesion extending from the left lumbar region to the epigastrium, with communication of the cyst with the midline laparotomy site (Figure 7). The pus aspirated from the wound site was collected and sent for culture and antibiotic sensitivity, and the presence of *E. coli* was identified.

After the initiation of appropriate culture-sensitive antibiotics (piperacillin with tazobactam intrave at 3.5 g thrice daily), the wound discharge eventually decreased over a period of 10 days. The stitching was removed on postoperative day 15, and the patient was discharged on postoperative day 20. The patient was on follow-up for three months and recovered without further issues.

## Discussion

Mesenteric cysts can occur anywhere in the mesentery and are most often seen adjoining the small intestine (40%) and colon (60%). There are four types of mesenteric cysts: chylolymphatic, enterogenous, traumatic, and hydatid cysts. The most common type is chylolymphatic, which occurs when congenitally malformed lymphatic tissue with no efferent lymphatic connection is present in the mesentery (mostly in the ileum). Chylolymphatic cysts have clear lymph or chyle content and also have a blood supply separate from the adjacent intestine. Conversely, enterogenous cysts are believed to be derived either from a diverticulum of the mesenteric border of the intestine that has been sequestered from the intestinal canal during the embryonic period or from a duplication of the intestine. The content of such cysts is mucinous and either colorless or brown due to past hemorrhage. These cysts have a common blood supply with the bowel, and hence, bowel resection is needed during cyst enucleation [2].

In terms of treatment, partially resected cysts may recur, and patients may present again with similar complaints. However, with large cysts, marsupialization may be the only option to salvage a large chunk of the bowel and prevent short-bowel syndrome [3].

Regarding previous research, according to Bolivar-Rodriguez et al., a 49-year-old male patient had abdominal pain and felt a mass over the abdomen. The patient was operated on, and an infected mesenteric cyst was removed. This research shows that such cysts can endanger life if not immediately removed [4].

Moreover, abdominal pain due to a mesenteric cyst may mimic appendicitis in non-pregnant females, as shown by Ward et al. Such a situation may produce a diagnostic dilemma, and thus, mesenteric cysts must be considered as one of the differential diagnoses when evaluating these cases [5]. Another case of mesenteric cysts presented by Bhattacharjee et al. in an elderly patient with abdominal pain and constipation highlights the necessity of noting mesenteric cysts as a potential differential diagnosis when evaluating abdominal pain across all age groups [6].

A case of acute abdomen from Kim et al. demonstrated that an infected mesenteric cyst may present as abdominal pain with a mass, requiring appropriate excision of the bowel with enucleation of the cyst for complete management [7].

## Conclusions

Most patients with mesenteric cysts are asymptomatic, and these cysts are often incidentally detected during regular imaging. An uncommon consequence, namely mesenteric cyst infection, is described in this case study. This work justifies performing urgent surgery to remove the cyst. In cases of difficult-to-excise mesenteric cysts that carry a risk of small bowel syndrome, marsupialization may be explored. Even if there is a chance that the cyst can recur, it is preferable to proceed with marsupialization due to the increased mortality and morbidity associated with short bowel syndrome. To ascertain the optimal course of treatment, it is also imperative to submit the cyst wall and cyst contents for histological and microbiological examinations. Overall, it is essential to properly diagnose mesenteric cysts and note them as a potential differential diagnosis to accurately manage such patients. 
